# Two Models for Time-Domain Simulation of Hybrid Magnetic Bearing’s Characteristics

**DOI:** 10.3390/s22041567

**Published:** 2022-02-17

**Authors:** Dawid Wajnert, Bronisław Tomczuk

**Affiliations:** Department of Electrical Engineering and Mechatronics, Opole University of Technology, PL-45758 Opole, Poland; b.tomczuk@po.edu.pl

**Keywords:** hybrid magnetic bearings (HMBs), simulation models of dynamics, magnetic equivalent circuit, finite element analysis, magnetic bearing transient states

## Abstract

A comparison of two developed simulation models for a hybrid magnetic bearing (HMB) transient states is presented. This applies to analyses using the flux-circuit directly coupled magnetic equivalent circuit and field-circuit indirectly coupled finite element analysis. The required control system was implemented for both models. The results obtained from the simulations were compared with those obtained from measurement tests.

## 1. Introduction

Magnetic bearings use the magnetic field to levitate the rotor without mechanical contact. They can be classified into three groups: active magnetic bearings (AMBs) [1], passive magnetic bearings (PMBs) [2] and hybrid magnetic bearings (HMBs) [3]. Magnetic bearings constitute a viable alternative for other types of bearings due to their unique properties, such as:

- the rotor of the bearing can rotate at very high speeds. The maximum rotational speed is limited by the critical speed of the rotor and stable rotation of the rotor achieved by the control system,
- magnetic bearings generate low losses, caused mainly due to eddy currents and hysteresis in the magnetic material of the stator and rotor. Additionally, at very high speeds, the losses are also caused by friction between the rotor surface and the air. The losses in magnetic bearings are 5 to 20 times lower than in conventional bearings at high speed [4], which significantly reduces operating costs,
- magnetic bearings are lubricant-free; therefore, they do not require sealings, nor a lubrication system, which reduces their maintenance costs,
- magnetic bearings are free of contamination wear, which makes them ideal for use in clean and sterile environments,
- the load of the bearing is limited mainly by the size of the bearing. However, the bearing load also depends on the type of the magnetic material, as well as on the stator construction,
- vibrations of the rotor are isolated from the machine body. Additionally, vibrations of the rotor can be actively suppressed by the control system by adjusting the stiffness and dumping factor of the bearing,
- the control system of the magnetic bearing allows for the easy implementation of the online diagnostics for the electric machine because it can access the position of the rotor and control currents. These signals can be used to determine the operating conditions and performance of the rotating machine,
- magnetic bearings can compensate unbalanced forces of the rotor by the control system,
- the lifetime of the magnetic bearings is almost unlimited because it operates contactless.

The important disadvantage of active/hybrid magnetic bearings is the need for applying the complicated control system because these types of magnetic bearings are inherently unstable devices. The control system includes regulators for the rotor position along the *x*- and *y*-axis. Dynamic responses of the HMB system constitute an important step during the process of the control system design. The often-used models for the transient response of magnetic bearings are based on equations that neglect the phenomena occurring in the magnetic material of the stator and rotor [5,6]. The more precise transient response of the control system can be obtained from models that take into account nonlinearity and saturation of the magnetic material, as well as leakage of the magnetic flux and eddy currents. This is especially important for hybrid magnetic bearings because their permanent magnets cause partial saturation of the magnetic circuit. Until now, various simulation models dedicated to transient characteristics of electric machines have been used. They include nonlinear *B*-*H* curve of the magnetic material, magnetic flux leakage and eddy currents. Four various models can be mentioned: field-circuit directly or indirectly coupled finite element models and flux-circuit directly or indirectly coupled magnetic equivalent circuits.

Field-circuit directly coupled finite element model (FC-FEM) is often named as a time-stepping finite element model [7,8]. This model couples magnetic field equations, external electric circuit equations and torque/force balance equations to simulate the transient performance of electric machines. Magnetic field distribution inside the electric machine has been calculated by 2D [7,8] and 3D FEM [9]. An external electric circuit can include an inverter supply system [10], distorted voltage-excited sources [9] and even control loops [11]. Commercially available software such as Ansys Maxwell or Comsol allows for simulating transient states based on FC-FEM [11,12]. Unfortunately, the calculation of transient states using FC-FEM takes a lot of CPU time, because magnetic field equations need to be solved at every simulation step of the circuit equations and torque/force balance equations.

The second type of simulation model is a field-circuit indirectly coupled finite element model. For this model, the magnetic field parameters, such as magnetic forces/torques, flux linkages, dynamic inductances and electromotive forces, are calculated beforehand from the finite element model (FEM). For this purpose, the 2D and 3D finite element models can be used. Afterwards, calculation results are incorporated into the simulation model as lookup tables [13,14,15]. Similarly to the previously presented dynamic simulation model, external electric circuit equations can include various supply systems, as well as control loops [13].

The third type of simulation model is a flux-circuit directly coupled magnetic equivalent circuit (FC-MEC). For this model, the magnetic field parameters of the electric machine are calculated directly from the magnetic equivalent circuit (MEC) at every solution of the external electric circuit equations and torque/force balance equations [16]. MEC of an electric machine can incorporate nonlinear characteristics of the magnetic material, as well as leakage and fringing fluxes [17,18]. A significant advantage of this simulation model in comparison to the field-circuit directly coupled FEM is the short time of calculations.

The last type of simulation model is a flux-circuit indirectly coupled magnetic equivalent circuit. For this model, the magnetic field parameters, such as magnetic forces/torques and flux linkages, are calculated beforehand from the magnetic equivalent circuit. Next, calculation results are integrated into the simulation model as lookup tables.

This paper aimed to present and compare two dynamic simulation models: FC-FEM and FC-MEC for the new construction of the hybrid magnetic bearing. Until now, there has been a lack of research papers that have demonstrated the dynamic simulation model for this construction of the HMB. Proposed models can be successfully used for testing different values of regulators’ parameters, as well as for simulating various working conditions. Responses obtained from simulation models were compared with measurements of the real object.

The paper is organized as follows: Section 2 describes the hybrid magnetic bearing that was analyzed in this study. Section 3 presents a description of two dynamic simulation models for the HMB, while Section 4 discusses simulation results compared with measurements. The paper finishes with conclusions presented in Section 5. Table 1 shows the list of acronyms used in the article.

## 2. Description of the Hybrid Magnetic Bearing

**Figure 1:**
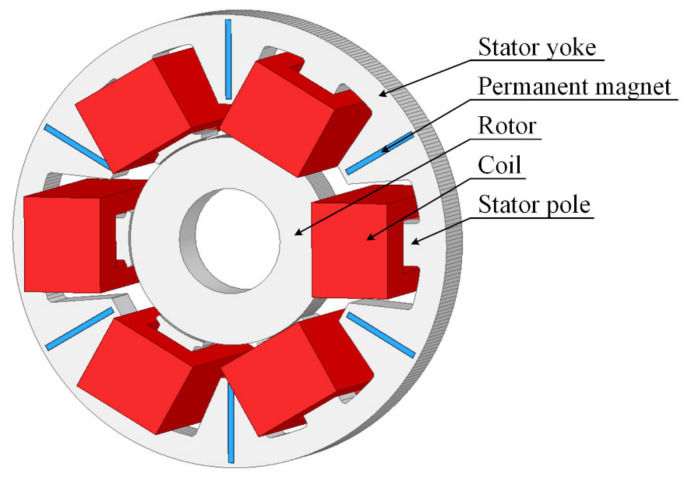
The geometry of the HMB magnetic circuit.

Figure 1 presents the geometry of HMB with three windings and six permanent magnets (PMs) installed in the stator yoke. One winding consisted of two coils, each of 100 turns; two coils were connected in series. Three windings generated the control flux for changing the magnetic force along the *x*- and *y*-axis. Permanent magnets N38 were installed in cut-out spaces of the yoke, which ensured precise fabrication of the stator. PMs provided the so-called bias flux that flows through all poles. Usage of permanent magnets reduces the consumption of electricity; therefore, hybrid magnetic bearings are dedicated to energy-saving devices. To significantly reduce the eddy current loss, the stator and rotor were manufactured from the laminated steel M400-50A. The stator outer diameter equaled 86 mm, while the stator inner diameter amounted to 40 mm. The rotor outer diameter equaled 39.4 mm; the air gap *δ* between the stator and rotor amounted to 0.3 mm. The stator length equaled 10 mm.

The principle of the presented HMB and 3D simulations of its magnetic field distribution are presented in [19]. Rated parameters of the HMB are listed in Table 2.

## 3. Dynamic Simulation Model

**Figure 2:**
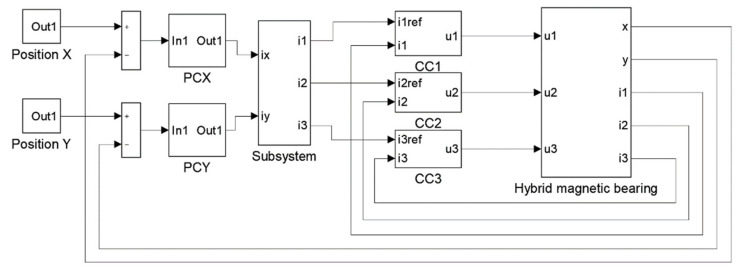
Block diagram of the HMB system.

The dynamic simulation model with the control system was implemented in MATLAB/Simulink software. Figure 2 presents connections between blocks in Simulink.

The control system contains three current control loops (CC1, CC2, CC3) and two position control loops (PCX, PCY). Current control loops stabilize required currents *i*_1_, *i*_2_, *i*_3_ in windings of the HMB. Values of these currents are determined by position controllers. The aim of position controllers is stabilization of the rotor in the middle of the stator. Parameters of the position controllers were calculated using the root locus method [20] while parameters of the current controllers were assumed to achieve fast dynamic response and acceptable overshooting.

The subsystem “Conversion” implements the conversion of the control currents into the currents excited in windings, which can be expressed according to the following formulas:

(1a) $$i_{1}=i_{y},$$

(1b) $$i_{2}=-\frac{1}{2}i_{y}+\frac{\sqrt{3}}{2}i_{x},$$

(1c) $$i_{3}=-\frac{1}{2}i_{y}-\frac{\sqrt{3}}{2}i_{x}.$$

The subsystem “Hybrid Magnetic Bearing” constitutes the implementation of the HMB dynamic simulation model. Two different dynamic simulation models were implemented into this subsystem: one based on FC-MEC and the second one based on FC-FEM. To precisely calculate magnetic flux linkages, as well as magnetic forces, both simulation models incorporate nonlinear characteristics of the magnetic material M400-50A. Due to the usage of laminated steel for the HMB construction, the simulation models have ignored the influence of eddy currents on the magnetic flux distribution.

### 3.1. Implementation of the Flux-Circuit Directly Coupled Magnetic Equivalent Circuit

The flux-circuit directly coupled magnetic equivalent circuit combines solutions from the magnetic model with ordinary differential equations (ODEs). The first type of ODEs describes the voltage drop across the stator windings:

(2) $$u_{k}=R_{k}i_{k}+\frac{d\Psi_{k}\left(x,y,i_{1},i_{2},i_{3}\right)}{dt},\;k=1\ldots 3,$$

where *u_k_* denotes winding supplying voltage, *i_k_* is the current excited in the winding, *R_k_* indicates the winding resistance and *Ψ_k_* denotes the magnetic flux linked with the *k*th winding. The value of flux linkage *Ψ_k_* is obtained directly from MEC.

The second type of equation concerns the movement of the rotor along the *x*- and *y*-axis. These equations are based on Newton’s second law of dynamics. It should be mentioned that the simulation model includes a static unbalance of the rotor and the gravity force acting along the *y*-axis:

(3a) $$m\frac{d^{2}x}{dt^{2}}=F_{x}\left(x,y,i_{1},i_{2},i_{3}\right)+m\omega^{2}e_{s}cos\left(\omega ,t\right),$$

(3b) $$m\frac{d^{2}y}{dt^{2}}=F_{y}\left(x,y,i_{1},i_{2},i_{3}\right)-mg+m\omega^{2}e_{s}sin\left(\omega t\right),$$

where *F_x_*, *F_y_* denote the magnetic forces. The symbol *ω* indicates the rotational speed, *e_s_* denotes the eccentricity, *m* indicates the mass of the rotor and *g* denotes the acceleration of gravity. For this simulation model, the flux linkages *Ψ*_1_, *Ψ*_2_, *Ψ*_3_ and the magnetic forces *F_x_* and *F_y_* are expressed as a function of the rotor position (in the *x*- and *y*-axis) and winding currents *i*_1_, *i*_2_, *i*_3_. Fixed-step solver ODE1 implemented in MATLAB/Simulink was used to solve the model and to obtain transient responses. The sample time of the model equals 50 µs.

**Figure 3:**
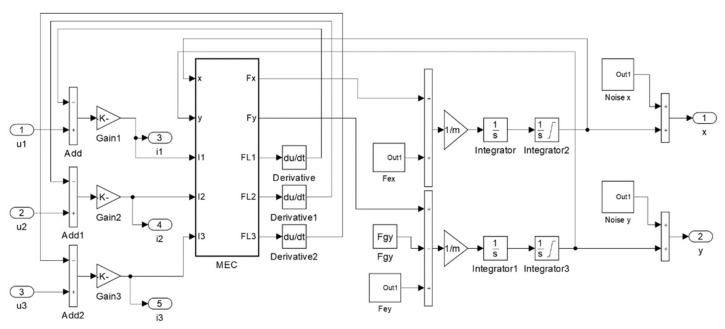
Implementation of the flux-circuit directly coupled magnetic equivalent circuit.

Figure 3 presents an implementation of FC-MEC prepared in Simulink software. The block “MEC” represents an implementation of the magnetic equivalent circuit for the analyzed HMB. Other blocks and connections constitute the implementation of equations describing voltage drop across windings (Equation (2)) and the rotor movement (Equation (3a,b)).

The magnetic equivalent circuit of HMB includes reluctances that represent basic components of the simulation model. The reluctances of the stator and rotor paths *R_µi_* were calculated from the expression:

(4) $$R_{µi}\left(B\right)=\frac{v\left(B\right)l_{i}}{A_{i}},$$

where *l_i_* denotes the length of an *i*th magnetic path, *A_i_* indicates the cross-section area of the magnetic path, *ν(B)* is the magnetic reluctivity for the dynamo steel sheets M400-50A.

The reluctances of permanent magnets *R_pm_* were calculated from the expression:

(5) $$R_{pm}=\frac{l_{pm2}}{{µ}_{0}{µ}_{rpm}l_{pm1}l_{s}},$$

where *l_pm_*_1_, *l_pm_*_2_ and *l_s_* indicate height, width and length of permanent magnets, respectively. Symbol *µ_rpm_* denotes relative magnetic permeability of permanent magnets and is equal to 1.0263. The reluctance of the *i*th air gaps *R_δi_* was calculated from the expression:

(6) $$R\delta_{i}=k_{c}\frac{\delta_{i}}{{µ}_{0}A_{AirGap}},$$

where *k_c_* denotes Carter’s factor, which was calculated from the 3D FEA, *A_AirGap_* is the cross-section area of the air gap and *δ_i_* indicates the length of air gaps between the stator poles and the rotor surface. The additional reluctance *R_µa_* that represents the leakage path of the flux in the proximity of permanent magnets was added to MEC.

The magnetic flux is provided by PMs and windings. The magnetomotive force (MMF) of permanent magnets is described by the following expression:

(7) $$F_{pm}=H_{c}l_{pm2},$$

where *H_c_* denotes the coercive force, while *l_pm_*_2_ indicates the width of permanent magnets. The MMF of the winding equals:

(8) $$F_{winding}=\lambda Ni,$$

where *λ* is the factor that takes account of the leakage effect of the winding, *N* indicates the number of turns and *i* is the current intensity that flows through the winding. The factor *λ* was calculated from the 3D FEA.

**Figure 4:**
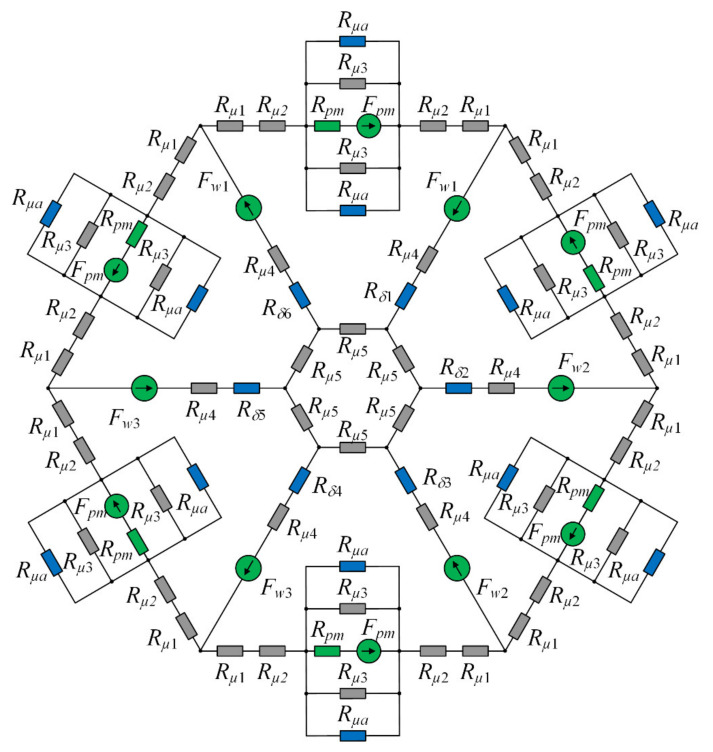
The magnetic equivalent circuit of the HMB.

In Figure 4 is presented a complete magnetic equivalent circuit for analyzed HMB.

Kirchhoffs’ laws for magnetic circuits were used to develop a set of nonlinear equations. 24 equations were derived from Kirchhoff’s flux law and 18 equations were obtained from Kirchhoff’s magnetic voltage/magnetomotive force law. The system of nonlinear equations is described by the following expression:

(9) $$f\left(\varphi \right)=A_{R}\varphi -F=0,$$

where *A_R_* denotes a matrix composed of reluctance elements, *φ* stands for a vector with the unknown branch fluxes and *F* denotes a vector with magnetomotive forces. The system of 42 nonlinear Equation (9) was solved by the iterative Broyden’s method [21]. The magnetic equivalent circuit was incorporated into the simulation model as a script for the MATLAB language that is invoked by Simulink for every step of the model solution. MEC of HMB contain five input parameters; three of them are currents *i*_1_, *i*_2_, *i*_3_ that flow through three windings and two of them are positions of the rotor along the *x*- and *y*-axis.

### 3.2. Implementation of the Field-Circuit Indirectly Coupled Finite Element Model

The field-circuit indirectly coupled finite element model is described by a similar set of ODEs for the electrical circuits and the mechanical system, as for the previous dynamic simulation model. However, the voltage drop across the windings is defined using dynamic inductances and velocity-induced voltages. These equations are expressed as:

(10) $$u_{k}=R_{k}i_{k}+L_{dk}\left(s_{k},i_{k}\right)\frac{di_{k}}{dt}+e_{vk}\left(s_{k},i_{k}\right)\frac{ds_{k}}{dt},\;k=1\ldots 3,$$

where *L_dk_* denotes the dynamic inductance of the winding, *e_vk_* indicates the velocity-induced voltage and *s_k_* denotes the position of the rotor along the three axes. The motion along the *x*- and *y*-axis of the rotor is determined by the following equations:

(11a) $$m\frac{d^{2}x}{dt^{2}}=F_{x}\left(x,i_{x}\right)+F_{x}\left(y,i_{y}\right)+m\omega^{2}e_{s}cos\left(\omega t\right),$$

(11b) $$m\frac{d^{2}y}{dt^{2}}=F_{y}\left(x,i_{x}\right)+F_{y}\left(y,i_{y}\right)-mg+m\omega^{2}e_{s}sin\left(\omega t\right),$$

where *F_x_* and *F_y_* denote magnetic forces along the *x*- and *y*-axis.

**Figure 5:**
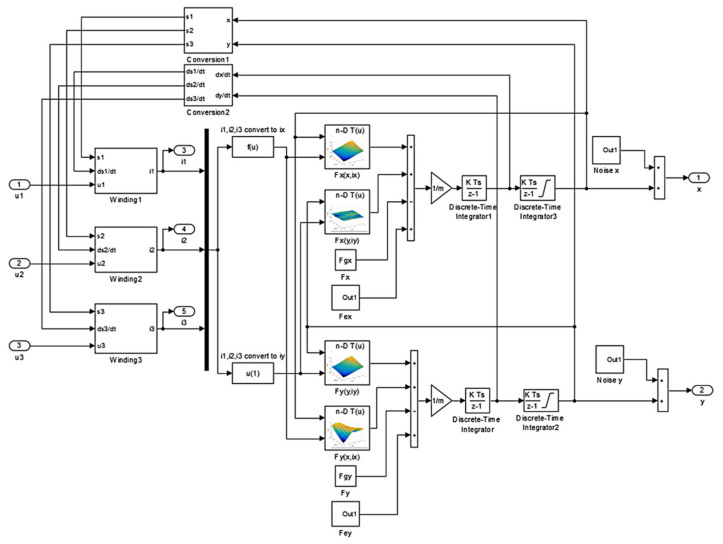
Implementation of the field-circuit indirectly coupled finite element model.

Figure 5 presents an implementation in Simulink of FC-FEM based on Equations (10) and (11a,b). Fixed-step solver ODE1 was used to solve the model and to obtain transient responses. The sample time of the model equals 50 µs. Calculated from 3D FEM parameters such as magnetic forces dynamic inductances and velocity-induced voltages were implemented into FC-FEM as look-up tables.

**Figure 6:**
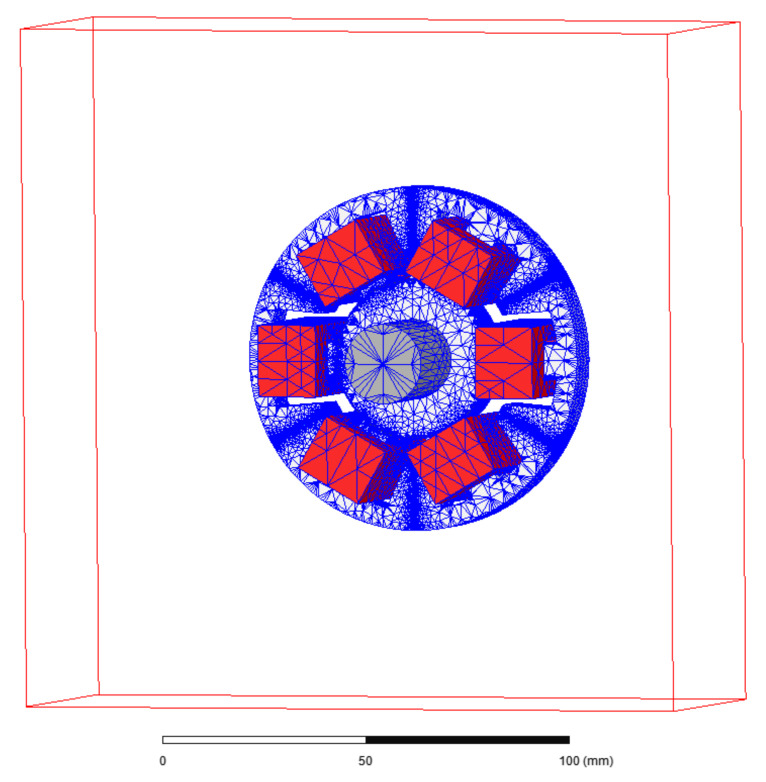
Finite element mesh of the simulation model.

Figure 6 depicts the simulation model with finite element mesh that was prepared using Ansys Maxwell 3D software.

**Figure 7:**
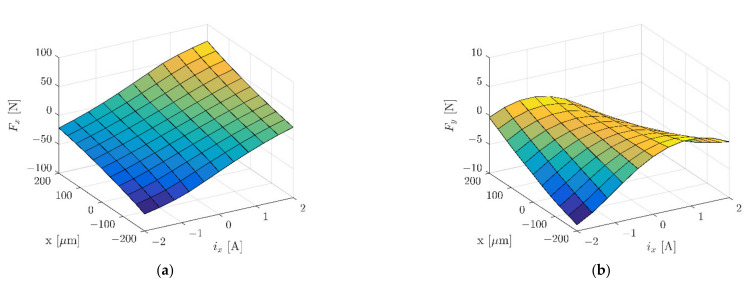
Magnetic force *F_x_* (**a**) and magnetic force *F_y_* (**b**) as a function of the rotor position *x* and control current *i_x_*.

**Figure 8:**
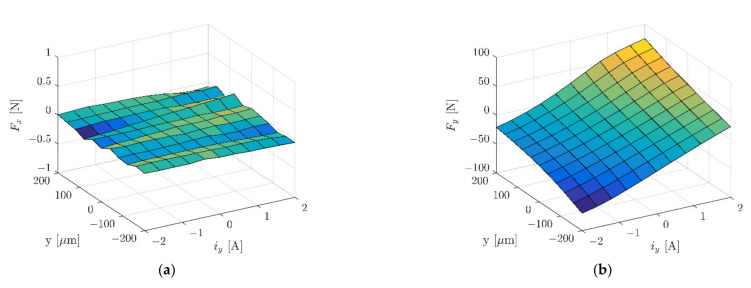
Magnetic force *F_x_* (**a**) and magnetic force *F_y_* (**b**) as a function of the rotor position *y* and control current *i_y_*.

**Figure 9:**
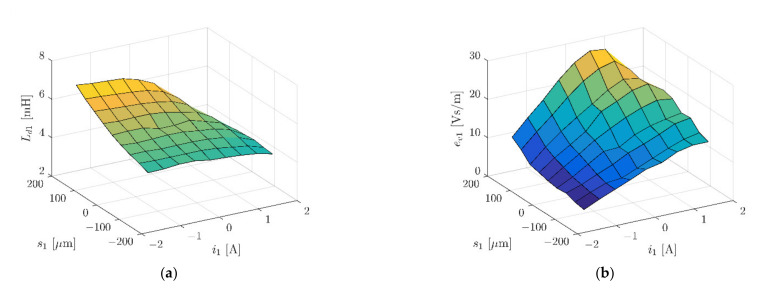
Dynamic inductance *L_d_*_1_ (**a**) and velocity-induced voltage *e_v_*_1_ (**b**) as a function of the rotor position *s*_1_ and winding current *i*_1_.

To limit the calculation time, the simulation model represented half of the real object. Further simplification of the model included the simple geometry of coils. The zero Dirichlet boundary condition for the magnetic field intensity was set to 40 mm from the stator in every direction. The symmetry plane was placed in the middle of the stator’s length with a zero Neumann boundary condition for the magnetic field intensity. Figure 7 and Figure 8 present magnetic forces *F_x_* and *F_y_*, while Figure 9 depicts dynamic inductance and velocity-induced voltage. All these parameters were incorporated into the simulation model presented in Figure 5.

## 4. Simulation Results

**Figure 10:**
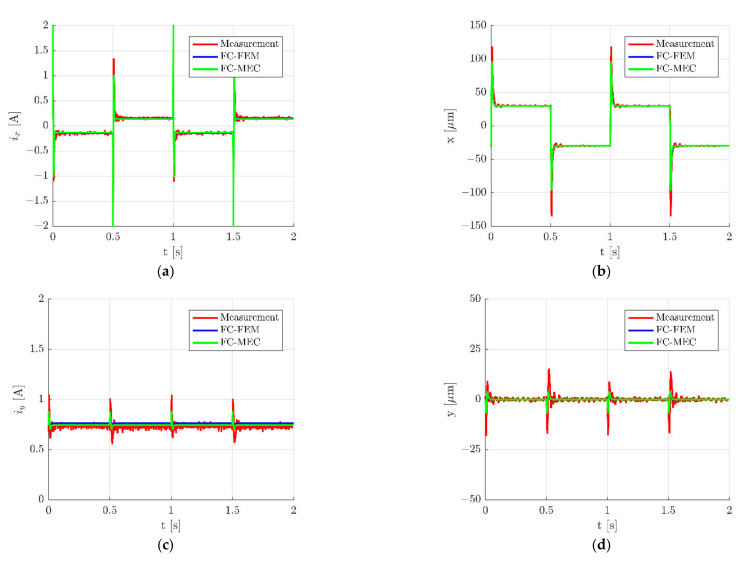
Time responses of the control current *i_x_* (**a**), the rotor position *x* (**b**), the control current *i_y_* (**c**) and the rotor position *y* (**d**) for the step change ±30 µm of the rotor position along the *x*-axis.

**Figure 11:**
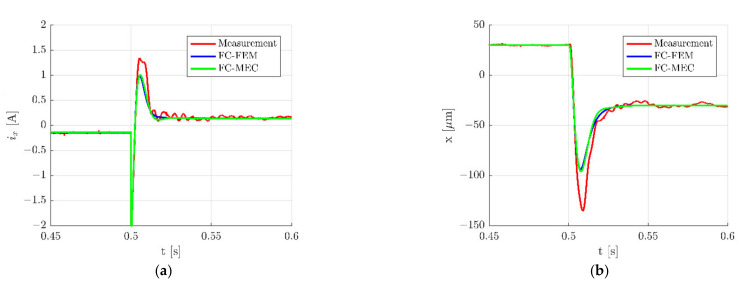
Zoom of time responses of the control current *i_x_* (**a**) and the rotor position *x* (**b**) for the step change ±30 µm along the *x*-axis.

Described simulation models were used to calculate dynamic responses of the HMB. Time responses during step change ±30 µm of the rotor position along the *x*-axis are presented in Figure 10, while zoomed waveforms of the control current *i_x_* and the rotor position along the *x*-axis are presented in Figure 11. These figures indicate a good agreement between results obtained from simulation models and measurements of the real object. However, it should be noted that measured signals possess higher overshooting (Figure 11), as well as contain interferences. Interferences of the control currents are caused by an electronic power supply that works with the frequency of 20 kHz.

**Figure 12:**
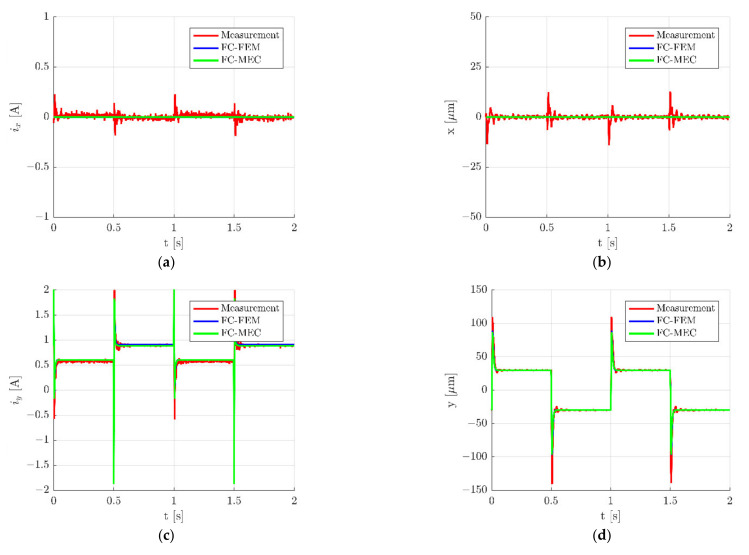
Time responses of the control current *i_x_* (**a**), the rotor position *x* (**b**), the control current *i_y_* (**c**) and the rotor position *y* (**d**) for the step change ±30 µm of the rotor position along the *y*-axis.

**Figure 13:**
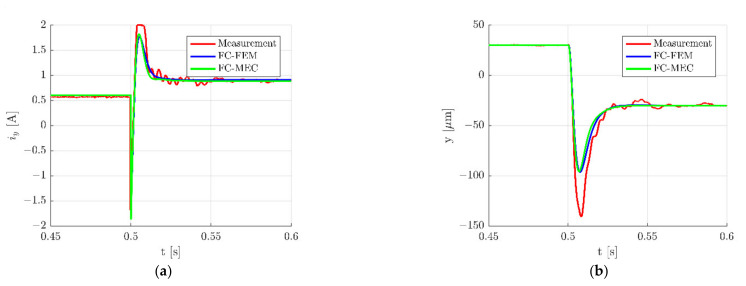
Zoom of time responses of the control current *i_y_* (**a**) and the rotor position *y* (**b**) for the step change ±30 µm along the *y*-axis.

The results obtained for the *y*-axis are presented in Figure 12 and Figure 13.

The accuracy of the simulation models was assessed by the calculations of the root mean square errors (*RMSEs*) between the measurement and simulation results from equations:

(12) $$RMSE_{x}=\sqrt{\frac{1}{n}\overset{n}{\underset{k=1}{\sum }}{\left(x_{mea}\left(k\right)-x_{sim}\left(k\right)\right)}^{2}}$$

(13) $$RMSE_{i_{x}}=\sqrt{\frac{1}{n}\overset{n}{\underset{k=1}{\sum }}{\left(i_{x-mea}\left(k\right)-i_{x-sim}\left(k\right)\right)}^{2}}$$

(14) $$RMSE_{y}=\sqrt{\frac{1}{n}\overset{n}{\underset{k=1}{\sum }}{\left(y_{mea}\left(k\right)-y_{sim}\left(k\right)\right)}^{2}}$$

(15) $$RMSE_{i_{y}}=\sqrt{\frac{1}{n}\overset{n}{\underset{k=1}{\sum }}{\left(i_{y-mea}\left(k\right)-i_{y-sim}\left(k\right)\right)}^{2}}$$

where *n* denotes the number of measurement points. The index *mea* indicates the quantities that were measured, while the index *sim* indicates the quantities that were simulated. In Table 3 and Table 4 are listed values of RMSEs for a step change ±30 µm of the rotor movement along the *x*- and *y*-axis, respectively. It can be seen that *RSMEs* calculated for both models hold similar values and that differences between them are insignificant. The only significant difference between *RSMEs* occurred for the control current *i_y_* (*RSME_iy_*) for a step change of the rotor along the *x*-axis (Table 3). The value of this error was by 18.20% less than that from FC-MEC in comparison to FC-FEM. This significant difference was caused by various characteristics of the magnetic force obtained from FEM and MEC.

It should be underlined that errors calculated for the control current *i_x_* (RMSE*_ix_*) and the rotor position *x* (RMSE*_x_*) for the step change of the rotor along the *x*-axis (Table 3) showed very similar values to errors calculated for the control current *i_y_* (RMSE_iy_) and the rotor position *y* (RMSE_y_) for the step change of the rotor along the *y*-axis (Table 4). This indicates that simulation models give similar dynamic responses for both axes similar to the real object.

**Figure 14:**
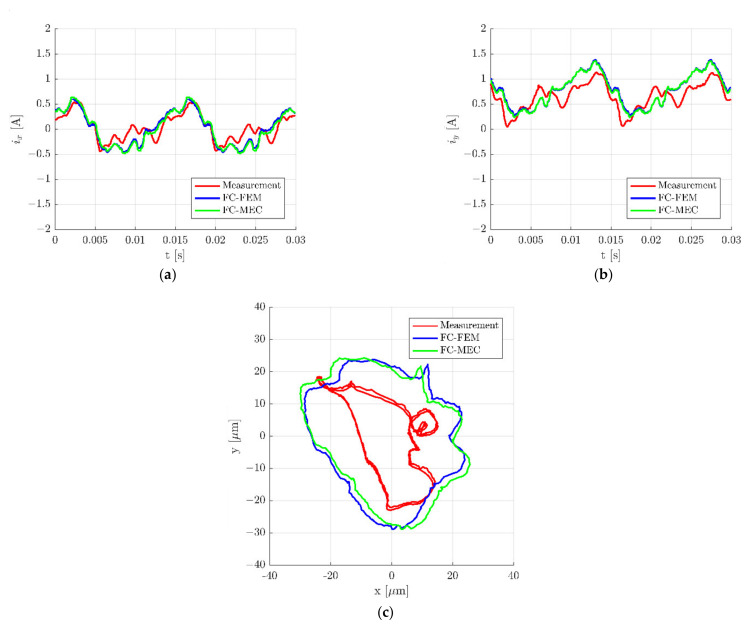
Time responses of the control currents *i_x_* (**a**) and *i_y_* (**b**) as well as the position of the rotor along the *x*- and *y*-axis (**c**) during rotation of the rotor with the speed 4123 rev/min.

Figure 14 presents time responses during rotation of the rotor with a speed of 4123 rev/min. It can be noticed that the results obtained from both simulation models are almost the same (blue and green lines). Simultaneously, repetitive variations of control currents signals occur in results obtained from simulations and measurements.

The higher discrepancy between simulations and measurements of the real object is visible for the rotor position along the *x*- and *y*-axis (Figure 14c).

## 5. Conclusions

The paper presents a comparison of two developed simulation models dedicated to calculation of transients for the hybrid magnetic bearing. These models are based on the field-circuit method, which includes voltage drop across windings, the rotor motion and the control system. For the first model, field parameters were obtained from the finite element analysis, while, for the second one, the field parameters were obtained from the magnetic equivalent circuit. Time responses of control currents and rotor positions were investigated during step change of the rotor position, as well as during rotation of the rotor. The obtained results showed good correlations between simulation results and measurements. The presented simulation models concerned a straightforward model for the rotor movement; therefore, it is recommended for electric machines with a fully levitating rotor to simulate the rotor dynamics as the rigid shaft or, in a more complicated situation, as the flexible shaft. Such an approach requires simulating magnetic bearings on both sides of the rotor.

To achieve higher rotational speeds of magnetic bearings, it is necessary to develop not only more accurate models for simulating mechanical characteristics but also models for simulation of heat flow (temperature distribution). This is especially true for HMBs, for which there are no standards that define the allowable temperature rise. In addition, although the experimental tests are expensive, the operating temperatures need to be verified, especially concerning the application of the different types of permanent magnets. Some of the problems given above will be researched in the future.

## Figures and Tables

**Table 1:** List of acronyms used in the article.

Abbreviation	Full Form
CC	Current controller
FC-FEM	Field-circuit indirectly coupled finite element model
FC-MEC	Flux-circuit directly coupled magnetic equivalent circuit
FEA	Finite element analysis
FEM	Finite element method
HMB	Hybrid magnetic bearing
MEC	Magnetic equivalent circuit
ODE	Ordinary differential equation
PCX	Position controller for the *x*-axis
PCY	Position controller for the *y*-axis
PM	Permanent magnet
RSME	Root square mean error
*A*	Cross-section area of the magnetic path
$e_{v}$	Velocity-induced voltage
$e_{s}$	Eccentricity
$F_{x}$ $,\;F_{y}$	Magnetic force for the *x*- and *y*-axis
$F_{maxx}$ $,F_{maxy}$	Maximal force for the *x*- and *y*-axis
$F_{pm}$	Magnetomotive force of the permanent magnet
$F_{w1}$ $,F_{w2}$ $,F_{w3}$	Magnetomotive forces of windings
$F_{0x}$ $,\;F_{0y}$	Initial force for the *x*- and *y*-axis
*g*	Gravitational acceleration
$H_{c}$	Coercive force of the permanent magnet
$i_{1}$ $,\;i_{2}$ $,\;i_{3}$	Currents of the windings
$k_{c}$	Carter’s factor
$k_{ix}$ $,k_{iy}$	Position stiffness
$k_{sx}$ $,k_{sy}$	Current stiffness
*l*	Length of the magnetic path
$L_{d}$	Dynamic inductance
*m*	Mass of the rotor
*N*	Winding number of turns
$R_{pm}$	Reluctance of the permanent magnet
$R_{1}$ $,\;R_{2}$ $,\;R_{3}$ $,\;R_{4}$ $,\;R_{5}$ $,\;R_{6}$	Reluctances of air-gaps
$R_{µ1}$ $,\;R_{µ2}$ $,\;R_{µ3}$ $,\;R_{µ4}$ $,\;R_{µ5}$	Reluctances of stator and rotor paths
*x*, *y*	Position of the rotor along the *x*- and *y*-axis
*λ*	Leakage factor of the windings
*µ*	Magnetic permeability
*ν*(*B*)	Magnetic reluctivity
*Ψ*	Linkage flux
*ω*	Rotational speed of the rotor

**Table 2:** Rated parameters of the HMB.

Parameter	Value
Position stiffness in the *x*-axis, *k_sx_*	105.93 N/mm
Position stiffness in the *y*-axis, *k_sy_*	106.03 N/mm
Current stiffness in the *x*-axis, *k_ix_*	21.97 N/A
Current stiffness in the *y*-axis, *k_iy_*	22.00 N/A
Initial force in the *x*-axis, *F*_0*x*_	21.84 N
Initial force in the *y*-axis, *F*_0*y*_	22.53 N
Maximal force in the *x*-axis, *F_maxx_*	41.84 N
Maximal force in the *y*-axis, *F_maxy_*	46.09 N
Dynamic inductance, *L_d_*	5.27 mH
Velocity-induced voltage, *e_v_*	15.56 Vs/m

**Table 3:** RMSE values for a step change of the rotor along the *x*-axis

	*RMSE_x_*	*RMSE_ix_*	*RMSE_y_*	*RMSE_iy_*
FC-FEM	15.48 μm	215.8 mA	2.764 μm	48.85 mA
FC-MEC	15.79 μm	221.2 mA	2.723 μm	39.96 mA

**Table 4:** RMSE values for a step change of the rotor along the *y*-axis

	*RMSE_x_*	*RMSE_ix_*	*RMSE_y_*	*RMSE_iy_*
FC-FEM	1.620 μm	25.66 mA	15.09 μm	205.6 mA
FC-MEC	1.631 μm	25.76 mA	15.34 μm	207.1 mA
